# Concordance between patient-reported outcomes and CTCAE clinician-reported toxicities during outpatient chemotherapy courses: a retrospective cohort study in routine care

**DOI:** 10.1016/j.esmorw.2025.100127

**Published:** 2025-03-27

**Authors:** S. Huynh-Dagher, T.-A. Duong, C. Tournigand, E. Kempf, G. Lamé

**Affiliations:** 1 Laboratoire Génie Industriel, CentraleSupélec, Université Paris-Saclay, Gif-sur-Yvette, France; 2 Service de dermatologie générale et oncologique CHU Ambroise Paré, AP-HP, Boulogne-Billancourt, France; 3 Université Paris Est Créteil, Assistance Publique – Hôpitaux de Paris, Department of Medical Oncology, Henri Mondor and Albert Chenevier Teaching Hospital, Créteil, France; 4 Sorbonne Université, Inserm, Université Sorbonne Paris Nord, Laboratoire d’Informatique Médicale et d’Ingénierie des Connaissances pour la e-Santé, LIMICS, Paris, France

**Keywords:** patient-reported outcome measures, quality of life, drug-related side-effects and adverse reactions, neoplasms, toxicity, drug monitoring

## Abstract

**Background:**

Patient-reported outcomes (PROs) are increasingly used but often correlate poorly with physicians’ assessment of adverse events. Yet, discordance has been associated with poorer prognosis.

**Methods:**

Single-centre retrospective study of patient and physician assessment of five chemotherapy-induced toxicities (mucositis, nausea, vomiting, peripheral neuropathy, diarrhoea) in routine care. Electronic PRO (e-PRO)-Common Terminology Criteria for Adverse Events (CTCAE) questionnaires were administered between outpatient chemotherapy sessions, using an online telehealth program. PRO-CTCAE grades were converted to CTCAE scale using the published composite grade of PRO-CTCAE, and compared with CTCAE grading of toxicities by physicians. We calculated Cohen’s weighted Kappa and counted discordances on severe (CTCAE grade >2) and milder (grade 2) toxicities.

**Results:**

A total of 122 patients filled electronically 519 PRO-CTCAE e-questionnaires for which physicians had rated at least one corresponding CTCAE item. Agreement between patients’ composite grades of PRO-CTCAE and clinicians CTCAE grades was moderate for four symptoms, and fair for one (κ between 0.23 and 0.43). The incidence of serious toxicity varied across symptoms, from 0.22% to 2.50% of questionnaires. Both patient and clinician scored a severe toxicity in only one case. Among 2076 symptom assessments, we counted 27 disagreements on severe toxicities (1.30%).

**Conclusion:**

Patient–physician agreement is imperfect, but severe toxicities are rare, including in PROs. PRO-CTCAE and CTCAE remain complementary, and e-PRO-CTCAE tools help monitor symptoms.

## Highlights

• Compare patient- and physician-reported toxicities between chemotherapy sessions.
• Severe toxicities (CTCAE >2) were rare.
• Agreement on toxicities is moderate.
• PRO-CTCAE and CTCAE are complementary.

## Introduction

In patients with active cancer, symptom monitoring and management is essential, as chemotherapy-associated adverse events (AEs) are a frequent iatrogenic issue.^1^ Patient-reported outcomes (PROs) are now widely used in clinical trials and in routine practice. Electronic collections of PROs (ePROs) are increasingly used, with benefits to patient outcomes.^2^ Paper-based and ePROs provide consistent results.^3^

The agreement between clinicians’ and patients’ assessment of anticancer treatment AEs, however, is generally moderate at best.^4^^,^^5^ Yet, patient–physician disagreement on status and outcomes has been associated with poorer prognosis.^6^

The Common Terminology Criteria for Adverse Events (CTCAE) is the widely accepted method used by clinicians to identify and grade AEs in cancer care and clinical research. A PRO companion to CTCAE, PRO-CTCAE, has been developed to support patient assessment of AEs using a common terminology for AEs, and is already used in trials.^7^ Based on a consultation of physicians, a composite grade of PRO-CTCAE has been proposed, in order to provide a single grade (instead of up to three in the regular reporting of PRO-CTCAE) and a grading system more aligned with the logic of the CTCAE.

In this study, we retrospectively analysed the level of agreement between clinicians’ grading of AEs using CTCAE and patients’ composite grade of PRO-CTCAE, in routine care.

## Methods

This quantitative, monocentric, retrospective study conforming to the French MR-004 standard was approved by the Henri Mondor Institutional Review Board (IRB#00011558) on 7 June 2024 (ref 2024-195). Only the data that were strictly necessary and relevant regarding the objectives of the research were collected and analysed during this project. We followed Strengthening the Reporting of Observational Studies in Epidemiology (STROBE) guidelines to report this study.^8^

### Population and setting

In 2017, the department of medical oncology of Henri Mondor University Hospital (Créteil, France) rolled out an electronic PRO monitoring (PROM) programme, Onco’Nect® (Nouvéal, Irigny, France). We had previously shown that most of our patients were equipped for and positive towards the use of e-medicine tools,^9^ despite the digital divide.^10^ In this e-PROM programme, all patients getting outpatient chemotherapy are offered the possibility to use an online interface that allows them to communicate with the department. Patients who enrol are invited to fill out a PRO-CTCAE questionnaire (v1.0)^11^ 2 days before, and 2 days after each cycle of i.v. anticancer drug (chemotherapy and/or immunotherapy combination). Grade >2 AEs send an alert to the care team, and patients can also generate an ‘I don’t feel good’ alert at any time. In these cases, a nurse navigator is notified by an ‘orange’ or ‘red alert’ on the web-based interface, and through automatic e-mails. Patients can also use Onco’Nect® to chat with the clinical team. Before each chemotherapy session, patients consult with an oncologist who grades the toxicities experienced since the previous treatment following CTCAE.

In this retrospective study, all patients >18 years old who enrolled in the Onco’Nect® programme between 1 September 2018 and 31 January 2021 were eligible, regardless of their type of tumour or chemotherapy regimen, except patients on oral therapy. We collected data from three electronic sources. First, we used the chemotherapy prescription system (Chimio®, Computer Engineering, Paris, France) to identify the dates of chemotherapy administration and the treatment regimens. Second, we manually extracted information from the outpatient record system, where oncologists fill out consultation reports that include the evaluation of toxicities following CTCAE v5.0 for all outpatient chemotherapy sessions. Third, we analysed PRO-CTCAE questionnaires filled by patients and stored on the database of the Onco’Nect® app.

We collected manually the following information for each patient: age, sex, type of cancer, socioeconomic status, treatment received, number of questionnaires filled, answers to PRO-CTCAE questionnaires for selected symptoms (mucositis, nausea, vomiting, peripheral neuropathy, diarrhoea), and clinician’s CTCAE rating of toxicities for the same subset of symptoms. We selected this subset of symptoms because of their high prevalence among outpatient cancer patients undergoing chemotherapy. All patients who rated at least one PRO-CTCAE symptom in a questionnaire, with a corresponding CTCAE questionnaire at least partly filled by the clinician (i.e. at least one item completed), were eligible for inclusion.

### Statistical analysis

Direct comparison of PRO-CTCAE and CTCAE grading is not possible. First, since the aims of CTCAE and PRO-CTCAE differ, the scales do not align (e.g. for diarrhoea, the fifth and most severe level on the PRO-CTCAE questionnaire is ‘almost constant’ diarrhoea, whereas grade 5 diarrhoea is ‘death’ in CTCAE). Second, CTCAE requires only one score per symptom, whereas PRO-CTCAE can have between one and three attributes per symptom (e.g. frequency and severity in the case of vomiting). To overcome this problem, Basch et al.^12^ (2021) proposed a composite grade of PRO-CTCAE that provides a single grade and a grading system more aligned with the logic of the CTCAE. For each symptom, each possible combination of scores on sub-items of the PRO-CTCAE table is matched with a corresponding score on a scale similar to that of CTCAE.

We calculated the patient’s composite grade of PRO-CTCAE answers using Basch et al.’s conversion tables. Configurations of sub-items that were not covered by the conversion tables were excluded, because they referred to illogical combinations [e.g. a patient rating the severity of mucositis as zero (none) but then rating the interference of mucositis with daily activities above zero], suggesting mistyping.

After calculating the composite grades of PRO-CTCAE, we analysed the concordance with clinician’s CTCAE grading of toxicities. We measured agreement between patient and clinician grading using Cohen’s weighted kappa (with quadratic weights). Agreement was defined as poor for κ <0, slight for 0< κ <0.2, fair for 0.21< κ <0.4, moderate for 0.41< κ <0.6, substantial for 0.61< κ <0.8, and almost perfect for 0.81< κ <1.^13^

We then focused on severe toxicities. Following previous works,^14^ we considered a CTCAE score ≥3 as a serious event. To assess if patients and clinicians agreed on severe toxicities (grade >2 on CTCAE and on composite grade of PRO-CTCAE), we counted occurrences where patients and clinicians agreed or disagreed on a CTCAE score equal or superior to 3 for a given symptom.

Finally, we specifically analysed milder symptoms, graded at 2 in the CTCAE. These symptoms are not severe enough to require urgent action, but they often damage patients’ quality of life. Here again, we counted occurrences, agreements, and up- and down-grading.

We used Wilcoxon and Kruskal–Wallis tests for comparing questionnaire filling rates between patient categories. All analyses were carried using R version 4.3.2, with the package ‘psych’ v2.4.3.^15^

## Results

We included 122 patients, of whom 60 (49%) were women (Table 1). The median duration of follow-up per patient was 121 days. The median age was 62.7 years [interquartile range (IQR) 53.9-70.3 years]. Patients filled a median of 10 PRO-CTCAE questionnaires (IQR 1-22 questionnaires). Patients completed a median of 60% (IQR 11%-24%) of the PRO-CTCAE questionnaires they were asked to answer. A total of 29 patients (24%) did not fill any of the PRO-CTCAE questionnaires they were sent. We found no significant relationship between age, sex, or socioeconomic categories and the response rate (*P* = 0.44, *P* = 0.53, and *P* = 0.09, respectively).

**Table 1:** Patient characteristics

Number of patients	122
Sex, *n* (%)	
Men	62 (51)
Women	60 (49)
Age (years), median (IQR)	62.7 (53.9-70.3)
Socioeconomic categories, *n* (%)	
Higher managerial and professional occupations	37 (30.3)
Clerical support, service, and sales workers	30 (24.6)
Technicians and associate professionals	22 (18.0)
Building workers and plant operators	6 (4.9)
Small employers and own account workers	3 (2.5)
Agricultural workers	1 (0.8)
Others	3 (2.5)
Unknown	20 (16.4)
Marital status, *n* (%)	
In a relationship	87 (71.3)
Alone	22 (18.0)
Unknown	13 (10.7)
Cancer, *n* (%)	
Breast	26 (21.3)
Colorectal	20 (16.4)
Digestive (non-colorectal)	45 (36.9)
Prostate	9 (7.4)
Urologic (non-prostate)	14 (11.5)
Other	4 (3.3)
Unknown	4 (3.3)
Treatment type, *n* (%)	
Anthracycline-based regimen	8 (6.6)
Mono-immunotherapy (checkpoint inhibitor)	8 (6.6)
Other	3 (2.5)
Cisplatin-based regimen	3 (2.5)
Microtubule inhibitor ± anti-HER2 therapies	21 (17.2)
Double-drug, carboplatin-based regimen	4 (3.3)
Docetaxel ± anti-HER2 therapies	6 (4.9)
FOLFIRI ± bevacizumab	11 (9.0)
Triple-drug, oxaliplatin-based regimen	12 (9.8)
Double-drug, oxaliplatin-based regimen	24 (19.7)
Gemcitabine	11 (9.0)
LV5FU2s ± bevacizumab	4 (3.3)
Targeted therapies	7 (5.7)
Number of questionnaires filled per patient, median (IQR)	10 (1-22)
Response rate to proposed questionnaires, median (IQR)	76% (33%-98%)
Number of patients with no questionnaire filled, *n* (%)	29 (24)
Follow-up in days, median (IQR)	121 (36-320)

HER2, human epidermal growth factor receptor 2; IQR, interquartile range.

We reviewed 519 questionnaires for which we had at least partial completion of PRO-CTCAE by the patient and partial completion of CTCAE by the clinician. In another 120 instances, we had partial PRO-CTCAE data, but no corresponding CTCAE data. These cases were excluded from the analysis.

**Figure 1:**
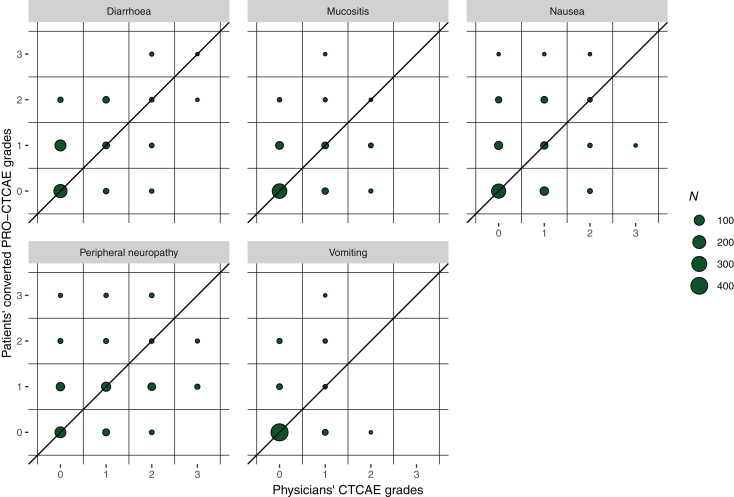
**Distribution of physicians’ CTCAE and patients’ converted PRO-CTCAE grades.** No grade above 3 was recorded. CTCAE, Common Terminology Criteria for Adverse Events; PRO, patient-reported outcome.

We obtained full PRO-CTCAE scoring between 360 and 460 times depending on the symptom (Table 2, Figure 1). Agreement between patients and clinicians was fair for four out of five symptoms, and moderate for one symptom. The incidence of serious toxicity varied across symptoms, from 0.22% to 2.5% of questionnaires. Peripheral neuropathy was the most frequent severe toxicity in both patients’ and clinicians’ responses.

**Table 2:** Grading of adverse events by patients and clinicians, by symptom. Patient-reported outcomes were converted from the PRO-CTCAE to the CTCAE scale.

Symptom	*N*	Patient score ≥3	Clinician score ≥3	Concordance		Discordance		Weighted Kappa (95% CI)
				Patient and clinician score ≥3	Patient and clinician score ≤2	Patient score ≥3 and clinician score ≤2	Patient score ≤2 and clinician score ≥3	
Mucositis	380	1 (0.26)	0 (0)	0 (0)	379 (99.74)	1 (0.26)	0 (0)	0.40 [0.28-0.51]
Nausea	456	3 (0.66)	1 (0.22)	0 (0)	452 (99.12)	3 (0.66)	1 (0.22)	0.31 [0.22-0.41]
Vomiting	460	1 (0.22)	0 (0)	0 (0)	459 (99.78)	1 (0.22)	0 (0)	0.23 [0.07-0.39]
Peripheral neuropathy	360	9 (2.50)	9 (2.50)	0 (0)	342 (95.00)	9 (2.50)	9 (2.50)	0.43 [0.35-0.51]
Diarrhoea	420	3 (0.71)	2 (0.48)	1 (0.24)	416 (99.05)	2 (0.48)	1 (0.24)	0.40 [0.30-0.50]
Total across symptom evaluations	2076	17 (0.82)	12 (0.58)	1 (0.05)	2048 (98.65)	16 (0.77)	11 (0.53)	

CI, confidence interval; CTCAE, Common Terminology Criteria for Adverse Events; PRO, patient-reported outcome.

Values expressed as number of questionnaires (percentage)

Disagreements on severe toxicity were rare, occurring in at most 2.5% of questionnaires (for peripheral neuropathy). Among all 2076 symptom assessments, we counted 27 disagreements on severe toxicity (1.3%).

Milder symptoms, graded at 2, varied in frequency (Table 3). Patients frequently declared nausea at grade 2 (9.9% of questionnaires), while clinicians often graded peripheral neuropathies at 2 (14.7% of questionnaires). Clinicians and patients varied in their assessment. When patients graded a symptom at 2, it was graded below 2 by clinicians in 66.7% (peripheral neuropathy, 12/18) to 100% (vomiting, 10/10) of cases. In reverse, when clinicians graded a symptom at 2, it had been graded below 2 by patients in 53.8% (diarrhoea, 7/13) to 100% (vomiting, 1/1) of cases.

**Table 3:** Clinicians’ scores when patients scored a symptom at 2, and patients’ scores when a clinician scored a symptom at 2

Symptom	*N*	Patient score = 2 (% total)	Clinician score (% patient scores)		
			=2	<2	>2
Mucositis	380	6 (1.6)	1 (16.7)	5 (83.3)	0 (0)
Nausea	456	45 (9.9)	4 (8.9)	41 (91.1)	0 (0)
Vomiting	460	10 (2.2)	0 (0)	10 (100)	0 (0)
Peripheral neuropathy	360	18 (5)	4 (22.2)	12 (66.7)	2 (11.1)
Diarrhoea	420	35 (8.3)	4 (11.4)	30 (85.7)	1 (2.9)


		Clinician score = 2 (% total)	Patient score (% clinician scores)		
			2	<2	>2
Mucositis	380	8 (2.1)	1 (12.5)	7 (87.5)	0 (0)
Nausea	456	13 (2.9)	4 (30.8)	8 (61.5)	1 (7.7)
Vomiting	460	1 (0.2)	0 (0)	1 (100)	0 (0)
Peripheral neuropathy	360	52 (14.7)	4 (7.7)	44 (84.6)	4 (7.7)
Diarrhoea	420	13 (3.1)	4 (30.8)	7 (53.8)	2 (15.4)

## Discussion

To improve symptom reporting in patients with cancer, patient-related outcomes have been developed in clinical trials and in clinical practice. The aim is to have a better reporting of a patient’s daily quality of life by asking the patient to fill the questionnaire directly without the physician's subjective interpretation. Furthermore, routine collection of PROs with electronic devices has demonstrated its positive impact on quality of life and survival, when combined with algorithms tracking symptom severity and alerting care teams or patients themselves.

Using a published conversion table to calculate composite grades of PRO-CTCAE and compare patient-reported toxicities and clinician-reported AEs, we found that regardless of the method used to compare them, agreement between patients’ and clinicians’ grading was moderate at best. Previous studies have found similar results, both in trial and routine care contexts.^4^^,^^14^^,^^16^ A systematic review identified frequent discordance between PROs and clinician-reported AEs in trials.^17^ In general, physicians underreport the prevalence and severity of patients’ symptoms, compared with direct reports by patients.^5^

Nonetheless, discordances on severe AEs (CTCAE ≥3) remained rare in our study. Grade 2 symptoms were also infrequent. Perhaps surprisingly, when grade 2 symptoms were reported by patients, physicians tended to downgrade them, but physicians also upgraded lower patient scores to a 2, especially for peripheral neuropathy, showing adjustments in both directions. Peripheral neuropathy is the symptom that received most grades at 2 or 3, both from patients and clinicians, but with frequent disagreements.

Several elements could explain the disagreements in grades. First, discordances could be explained by the fact that patient and clinician ratings happen at different points in time (in-between chemotherapy sessions for PRO-CTCAE, right before a session for CTCAE). PRO-CTCAE questionnaires use a recall period: questions are formulated as ‘In the past X days, have you experienced…?’. This recall period can slightly affect results.^18^ On this point, the rating of CTCAE in routine care left a margin of interpretation to the clinician, with no specific instruction to score the current level of toxicities, the apex over a period, or another metric. The frequency of questionnaires also affects patients’ reporting of toxicities.^19^ More fundamentally, the aims of PRO-CTCAE and CTCAE differ,^14^ and it should not come as a surprise that they provide complementary perspectives rather than duplicate the same assessment. Methodological aspects in our study may also have affected our results. The tables we used to calculate a composite grade of PRO-CTCAE to CTCAE were developed based on the input of clinical investigators solely (although patients participated in other aspects of the study).^12^ Optimistic assessment of efficacy and toxicity of anticancer treatment by oncologists compared with nurses and patients has been reported.^5^^,^^20^ It may have permeated in these tables, leading to an under-evaluation of the functional impact of side-effects on patients’ quality of life.

A strength of this study is the use of validated conversion tables, which allowed us to use dedicated, validated questionnaires for both patients and clinicians, while still permitting a direct comparison between scores. Besides, we used real-life data from routine care. Nonetheless, this study has limitations. First, it may reflect specific behaviour of physicians in this department. Second, previous research showed that clinician grading of AEs can be inconsistent,^21^ and we did not account for between-clinician variability in this study. Third, the matching strategy between PRO surveys and clinician surveys is sub-optimal, as only pairs of surveys with at least a symptom reported by the patient and the clinician were included, thus underrepresenting the scenarios where either patients or clinicians do not report a symptom that was present (under-reporting). This strategy artificially reduces false-negative cases and may overestimate concordance by eliminating false negatives by design. Fourth, some common symptoms, e.g. pain and fatigue, were not included, whereas their identification and graduation include a level of subjectivity that is amenable of discrepancy between patients and clinicians. This, again, might lead to overestimate agreement.

Actively involving cancer patients and oncology nurses in developing and refining composite grades of PRO-CTCAE could help improve the clinical relevance of these tools.^22^ Assessing if and how patient–clinician agreement varies between clinicians would also be useful, to understand if certain physician characteristics are associated with more frequent discordances. Finally, understanding why a significant subpopulation of cancer outpatients do not complete any ePRO questionnaires may improve the quality of their monitoring. Similar studies to the one reported here could also look into AEs related to oral treatments and radiotherapy, or to novel therapies deployed since 2021.

## Conclusion

In a population of cancer outpatients undergoing systemic therapy, the level of concordance on the severity of signs and symptoms between patients and clinicians was moderate at best. Discordances on severe AEs, however, remained rare. Self-evaluation by PRO-CTCAE remains useful to detect severe AEs.
